# Computational neurobiology is a useful tool in translational neurology: the example of ataxia

**DOI:** 10.3389/fnins.2015.00001

**Published:** 2015-01-21

**Authors:** Sherry-Ann Brown, Louise D. McCullough, Leslie M. Loew

**Affiliations:** ^1^Department of Medicine, Mayo ClinicRochester, MN, USA; ^2^Departments of Neurology and Neuroscience, University of Connecticut Health CenterFarmington, CT, USA; ^3^Richard D. Berlin Center for Cell Analysis and Modeling, University of Connecticut Health CenterFarmington, CT, USA

**Keywords:** spinocerebellar ataxia, translational, model, computational, neurology, inositol 1,4,5-triphosphate receptor 1, Purkinje, homer

## Abstract

Hereditary ataxia, or motor incoordination, affects approximately 150,000 Americans and hundreds of thousands of individuals worldwide with onset from as early as mid-childhood. Affected individuals exhibit dysarthria, dysmetria, action tremor, and diadochokinesia. In this review, we consider an array of computational studies derived from experimental observations relevant to human neuropathology. A survey of related studies illustrates the impact of integrating clinical evidence with data from mouse models and computational simulations. Results from these studies may help explain findings in mice, and after extensive laboratory study, may ultimately be translated to ataxic individuals. This inquiry lays a foundation for using computation to understand neurobiochemical and electrophysiological pathophysiology of spinocerebellar ataxias and may contribute to development of therapeutics. The interdisciplinary analysis suggests that computational neurobiology can be an important tool for translational neurology.

## Introduction

**Figure 1:**
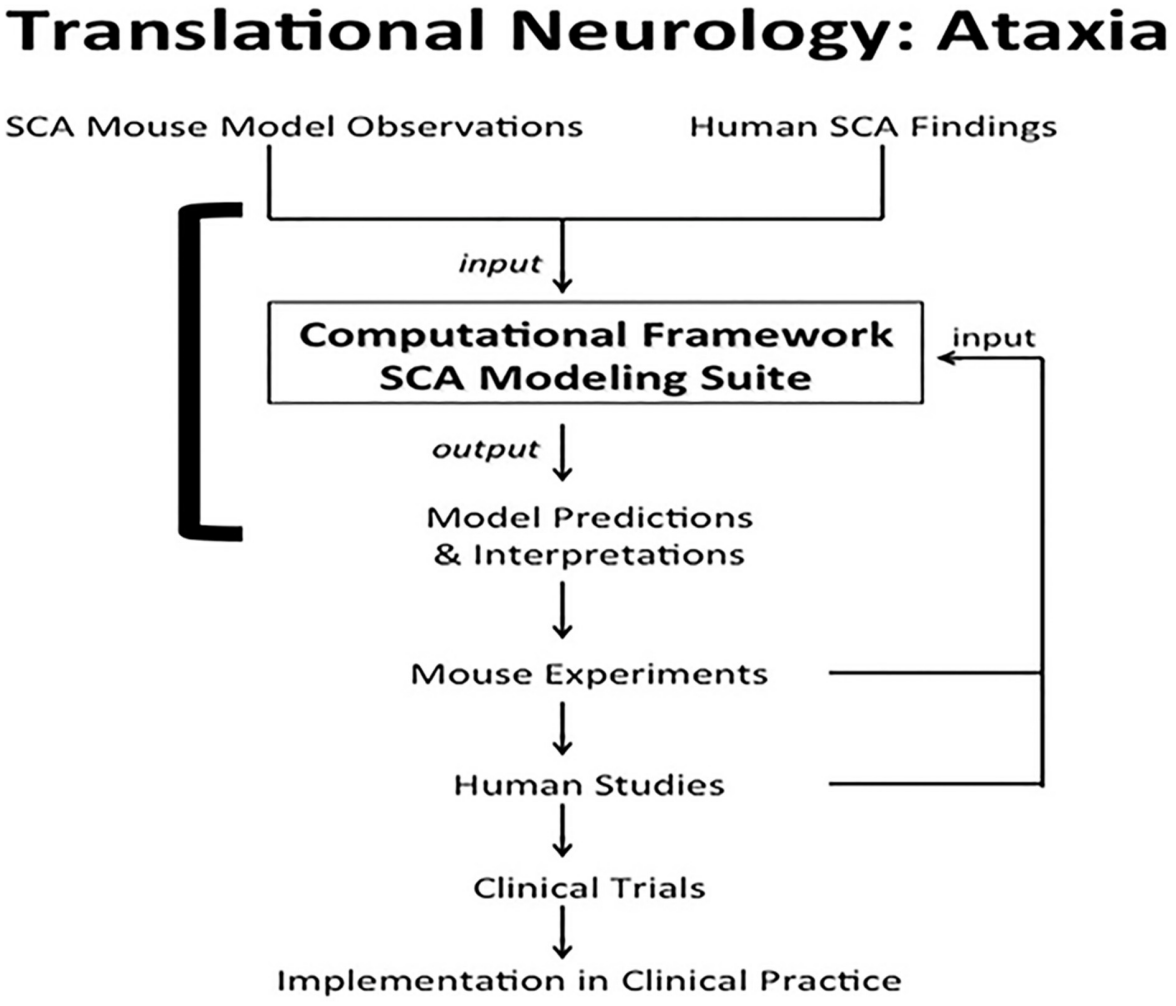
**Translational neurology: ataxia**. SCA mouse observations and human SCA findings are incorporated into the computational framework SCA modeling suite. The models help interpret experimental and clinical findings. The models also predict interactions between proteins and emergent properties that can be borne out in novel mouse experiments. Results from the laboratory and clinical observations can be used to validate, disprove, or tweak the computational models. Findings from mouse experiments can also ultimately be translated to human studies, leading to clinical trials to test therapeutics. The final step in translational neurology with the example of ataxia is implementation of the iterative findings in patient care. The solid square bracket highlights the components addressed directly by computational systems neurobiology (Brown et al., 2012; Brown and Loew, 2012).

Computational systems neurobiology (Brown et al., 2012) can be used to understand neuronal systems, based on utilizing information garnered from clinical reports, animal studies and *in vitro* modeling. Results from computational neurobiology can be used to develop additional animal and cellular experiments that may ultimately be translated to clinical practice, i.e., translational neurology. One clinical condition poised to benefit from this marriage is spinocerebellar ataxia (SCA) (Figure 1). Ataxia refers to lack of motor coordination (Goetz, 2003). In this paper, we use SCA as an example to demonstrate how computation and translation can potentially be woven together to enhance our knowledge of cell function.

The most common SCAs are spinocerebellar ataxia 1 (SCA1), spinocerebellar ataxia 2 (SCA2), spinocerebellar ataxia 3 (SCA3), and Spinocerebellar Ataxia 6 (SCA6) (Supplementary Material, Table S1) (Jacobi et al., 2012; Orr, 2012; Musova et al., 2013). They are caused by expanded polyglutamine (polyQ; CAG) repeat mutations in genes that code for ataxin 1, ataxin 2, ataxin 3, and the CACNA1A calcium channel, respectively (Supplementary Material, Table S1) (Gispert et al., 1993; Orr et al., 1993; Kawaguchi et al., 1994; Pulst et al., 1996; Zhuchenko et al., 1997; Tonelli et al., 2006; Bürk et al., 2014).

A number of mutations that cause SCA or episodic spinocerebellar ataxia (EA) also occur in genes directly involved in calcium signaling and plasma membrane excitability, which are critical for cerebellar Purkinje neuron function (Kim et al., 1997; Yue et al., 1997; Zhuchenko et al., 1997; Guida et al., 2001; Alonso et al., 2005; Iwaki et al., 2008; Becker et al., 2009; Alviña and Khodakhah, 2010; Kasumu and Bezprozvanny, 2012) (Supplementary Material, Table S1). A number of these genes impact the phosphoinositol signaling pathway (Supplementary Material, Figure S1A; Table S1) (Aiba et al., 1994; Kim et al., 1997; Alonso et al., 2005; van de Leemput et al., 2007). This pathway is important for calcium release from the smooth endoplasmic reticulum (sER) into the cytoplasm of cerebellar Purkinje neurons. Many other mutations affect calcium and potassium ion channels (EA2, SCA6, EA1, EA5, SCA13, SCA19, SCA22) (Supplementary Material, Table S1) (Yue et al., 1997; Zhuchenko et al., 1997; Lin et al., 2000; Guida et al., 2001; Imbrici et al., 2003; Sausbier et al., 2004; Tonelli et al., 2006; Bürk et al., 2014) that are important for regulating the rate of calcium influx into cells. spinocerebellar ataxia 14 (SCA14) involves a mutation in the gene encoding protein kinase C (PKC) that is also important for calcium homeostasis (Supplementary Material, Figure S1A; Table S1) (Alonso et al., 2005; Ueda et al., 2013; van Gaalen et al., 2013; Ji et al., 2014). spinocerebellar ataxia 15 (SCA15) and spinocerebellar ataxia 16 (SCA16) in humans and in mice are caused by deletion and missense mutations in the gene for inositol-1,4,5-trisphosphate receptor type 1 (IP3R1), a calcium channel on the sER (Desaiah et al., 1991; Street et al., 1997; Zecevic et al., 1999; Lin et al., 2000; Storey et al., 2001; Serra et al., 2004; van de Leemput et al., 2007; Chen et al., 2008; Chou et al., 2008; Hara et al., 2008; Iwaki et al., 2008; Liu et al., 2009; Di Gregorio et al., 2010; Novak et al., 2010a,b; Huang et al., 2012).

Data from mouse experiments and clinical observations have been incorporated into SCA computational models (Brown and Loew, 2015). The models are developed using computer engineering tools and software, such as Virtual Cell (Moraru et al., 2008; Slepchenko and Loew, 2010) and NEURON (Hines and Carnevale, 2001). These modeling platforms are based on mathematical equations for physics principles, such as reaction, diffusion, flux, and concentration gradients (Hines and Carnevale, 1997; Schaff et al., 2000; Cowan et al., 2012). The models simulate physiological interactions among intracellular reactions, molecular diffusion, and cell geometry and give insight into how these cellular processes work together as an efficient system (Brown et al., 2008; Brown and Loew, 2012). Simulations help clarify observed phenomena and suggest future experiments to help elucidate disease mechanisms and potential therapeutics.

## Experiments confirm model predictions

There are a handful of neurobiological and electrophysiological computational models relevant to IP3R1-associated ataxias (Brown and Loew, 2012), detailed in the following subsections.

### Neurobiology modeling and simulation

#### PIP2 signaling upstream of IP3R1

First, a model of phosphatidylinositol-4,5-bisphosphate (PIP2) signaling upstream of IP3R1 in neuroblastoma cells was developed (Xu et al., 2003) (Supplementary Material, Figure S1A). The model predicted stimulated PIP2 synthesis in addition to PIP2 hydrolysis when the Purkinje neuron spine is activated by parallel fibers, to produce IP3R1-mediated calcium release. Subsequently, bench experiments confirmed simulation results in a mouse neuroblastoma cell line (Xu et al., 2003).

#### IP3R1 signaling downstream of PIP2

Second, models of IP3R1-related signaling downstream of PIP2 in the cerebellar Purkinje neuron were created (Doi et al., 2003; Hernjak et al., 2005). One model considered the high abundance and low sensitivity of IP3R1 in Purkinje cells (Hernjak et al., 2005). This model qualitatively reproduced experimentally observed calcium transients during coincident activation of the Purkinje spiny dendrite (Wang et al., 2000).

#### Sources of sufficient PIP2 for IP3R1-mediated signaling

Third, quantitative models of Purkinje neurons spiny dendrites were developed (Brown et al., 2008, 2011; Brown and Loew, 2012). Local sequestration of PIP2 (with a lower diffusion coefficient than unbound PIP2) on the inner leaflet of cerebellar Purkinje neuron spines (McLaughlin et al., 2002; Golebiewska et al., 2008) (Supplementary Material, Figure S1B) was assessed. Model results supported the efficacy of local sequestration as a means of providing sufficient PIP2 for IP3R1-mediate calcium release. Findings correlated with prior experimental results (Wang et al., 2000) (Table 1). The computational simulations predicted a time window during which coincident activation of the Purkinje spine by other cell types could occur (Brown et al., 2008). This time window was independently borne out in bench experiments in rat cerebellar brain slices (Sarkisov and Wang, 2008) (Table 1).

**Table 1:** **Examples of contributions of computational systems neurobiology to translational neurology**.

**Mechanism elucidated**	**Computational prediction**	**Supporting experiments**	**References (Computational/Experiment)**
Source of requisite IP3	PIP2 synthesis concurrent with hydrolysis	IP3 production in neuroblastoma cells	Xu et al., 2003/Xu et al., 2003
Purkinje spine electrophysiology	D-type K and class-E Ca channels required	Purkinje neuron current clamp	Miyasho et al., 2001/Miyasho et al., 2001
Biochemical-electrical cross-talk	Emergent cross-signaling properties	Biochemical before electrical changes in SCA2 mice	Brown and Loew, 2012/Hansen et al., 2013
AMPAR all-or-none activation	MAPK-PKC positive feedback loop	Purkinje stimulation by CFs/PFs	Ogasawara et al., 2008/Ogasawara et al., 2008
Local PIP2 sequestration	Fine-tunes coincidence detection	Purkinje stimulation by CFs/PFs	Brown et al., 2008/Wang et al., 2000
Coincidence detection	50–100 ms time window CF before PF	Purkinje stimulation by CFs/PFs	Brown et al., 2008/Sarkisov and Wang, 2008
IP3R1 compensation	IP3R1 downregulation in polyQ disorders with IP3R1 supersensitivity	IP3R1 (and other members of the signaling complex^*^) downregulation in DLPRA	Brown and Loew, 2012/Suzuki et al., 2012

* *(Supplementary Material, S1 Signaling complex regulation)*.

#### Signaling downstream of IP3R1

Fourth, predictions were generated about kinetic interactions between PKC and α-amino-3-hydroxy-5-methylisoxazole-4-propionic acid subtype glutamate receptors (AMPAR) in Purkinje neurons that were experimentally verified (Ogasawara et al., 2008) (Table 1). These molecules are downstream of IP3R1-mediated calcium release, as calcium co-activates PKC.

#### Potential use of neurobiological model results

Using results from these computational models, drugs could be developed to interfere with local sequestration or other steps in the phosphoinositol signaling pathway in mice or rats and could potentially be translated to humans with supersensitive IP3R1 to help treat SCA1-3 and SCA14 (Supplementary Material, Table S1).

### Neuroelectrophysiology modeling

#### Prediction of purkinje neuron calcium and potassium channels

Normal electrophysiology of the Purkinje neuron was also modeled (De Schutter and Bower, 1994a,b; Miyasho et al., 2001). New properties of Purkinje neuron electrophysiology were predicted, requiring contributions of D-type potassium channels and class-E calcium channels that were not known previously to influence Purkinje neuron electrophysiology (Supplementary Material, Figure S1A). The predictions were confirmed with rat model experiments (Miyasho et al., 2001) (Table 1).

#### Reduction of purkinje neurons

A method to map realistic neurons into equivalent reduced models while maintaining high accuracy membrane potential changes during synaptic inputs, with direct links to experimental observables was subsequently developed (Marasco et al., 2012).

#### Potential use of neuroelectrophysiological model results

The examples provided suggest that iterative computational modeling can provide insight into normal and pathological neurophysiology. The neuroelectrophysiology models could be used to economically assess the impact of new therapeutics in research and development prior to studies in mice or rats. Manipulation in the virtual system would allow for precise input control and real-time output review with changes in firing dynamics of the Purkinje neuron. This would facilitate discovery of determinant and feedback loops (Brown and Loew, 2015) and other interactions that would otherwise be impossible to monitor in brain slices in the same time frame. Insights could potentially be translated to humans with various SCAs, particularly those such as SCA6, SCA13, SCA19, and SCA22 that involve disruption of membrane electrophysiology (Supplementary Material, Table S1).

## Simulations interpret observed phenotypes

### Supranormal IP3R1 sensitivity in SCA 1-3

A source of pathology caused by the polyQ repeats (Orr et al., 1993; Kawaguchi et al., 1994; Koide et al., 1994, 1999; Trottier et al., 1994; Pulst et al., 1996; David et al., 1997; Nakamura et al., 2001) is due to interaction of the mutant protein with IP3R1 (Bezprozvanny, 2011). In SCA2 (Liu et al., 2009) and SCA3 (Chen et al., 2008), mutant Ataxin-2 and Ataxin-3, respectively, directly bind to the C-terminal of IP3R1 and make it easier to solicit IP3-induced calcium responses. Association of mutant Ataxin-1 with IP3R1 has been reported (Liu et al., 2009), but supersensitivity in these mice has not yet been tested. SCA modeling results suggests that IP3R1 supersensitivity in SCA1 is necessary (Brown and Loew, 2012) to elicit observed supranormal calcium transients (Inoue et al., 2001).

### IP3R1 downregulation provides partial compensation in SCA 1-3

Studies in SCA1 and SCA2 mouse models, as well as mice and humans with SCA3, found reduced levels of IP3R1, metabotropic glutamate receptor (mGluR), and other calcium signaling and glutamatergic proteins (Lin et al., 2000; Vig et al., 2001; Serra et al., 2004; Chou et al., 2008; Hansen et al., 2013) (Supplementary Material, Figure S1). Decreased expression of IP3R1 and sarcoendoplasmic reticulum calcium ATPase (SERCA) was also confirmed in SCA1 patients (Lin et al., 2000). Such findings were also discovered in Purkinje neurons from mouse models of HD (Datta et al., 2011; Euler et al., 2012). SCA modeling interprets downregulation of these key calcium signaling proteins as serving to partially compensate for supersensitive IP3R1 (Brown and Loew, 2012). Further downregulation of these glutamatergic signaling proteins could be manipulated to delay symptomatic disease in mouse models and in the long run in presymptomatic humans (Brown and Loew, 2015).

### Homer and myosin VA association with IP3R1 in SCAs

Homer 3 is part of a signaling complex with reduced expression in SCA1 mice (Serra et al., 2004) (Supplementary Material, S1 Signaling complex regulation). Homer 3 localizes predominantly to Purkinje neuron spines (Shiraishi et al., 2004) and may associate with mGluR and IP3R1 *in vivo* (Tu et al., 1998; Sandonà et al., 2003). Myosin Va levels are also decreased in SCA1 (Serra et al., 2004). Both Homer and Myosin Va have been proposed to guide sER (sER) into spines as spines are being formed from dendritic shafts (Wagner and Hammer, 2003). Accordingly, Myosin Va knockout mice are ataxic with spines devoid of sER and IP3R1 (Takagishi et al., 1996). SCA modeling results suggest that reduced spine sER volume due to downregulation of Homer 3 and Myosin Va in SCA1 partially compensates for IP3R1 supersensitivity (Brown and Loew, 2012). This supports findings from an experiment in which downregulation of Homer 1b/c attenuated IP3R1-mediated calcium release in rat cortical neurons (Chen et al., 2012). Expression of Homer could potentially be manipulated to further compensate for IP3R1 supersensitivity in polyQ ataxias, which also include SCA7 (David et al., 1997), SCA17 (Nakamura et al., 2001), and dentatorubral-pallidoluysian atrophy (DLPRA) (Koide et al., 1994). SCA simulations could determine a therapeutic window for Homer expression to avoid overcompensation. As an example, although a very different mechanism, Homer 3 scaffolding protein has been implicated as an autoimmune target in subacute cerebellar ataxia. This ataxia is not hereditary and occurs post-infection or as a paraneoplastic process in some patients with Hodgkin's lymphoma (Zuliani et al., 2007). Presumably, disruption of Homer 3 scaffolding beyond a therapeutic window, or in the absence of supersensitive IP3R1 as in autoimmune subacute cerebellar ataxia, interrupts signaling complex formation and related cellular processes (Supplementary Material, S1 Signaling complex regulation).

### Cross-signaling between biochemical and electrophysiological dysregulation in SCAs

These and other forms of biochemical dysregulation precede electrophysiological impairment in an SCA2 mouse model, with Purkinje neuron firing frequency decreased at 6 weeks compared to wild type (Hansen et al., 2013) and worsening as the mice age (Kasumu et al., 2012b). This is consistent with timing of electrophysiological changes in an SCA1 mouse model (Hourez et al., 2011) and in the large conductance calcium-activated voltage-gated potassium channel (BK) knockout mice (Sausbier et al., 2004). These changes are then followed by onset of motor discoordination at 8 weeks in the SCA2 mice (Hansen et al., 2013) and 6–8 weeks in SCA3 mice (Shakkottai et al., 2011). The time of ataxia onset for these mice is identical to that for mice heterozygous for IP3R1 deletion (Ogura et al., 2001). These findings imply shared pathophysiology leading to similar phenotype: biochemical dysfunction and subsequent electrophysiological aberrations leading to ataxia. The SCA modeling suite has also predicted altered Purkinje neuron firing arising from cross-talk between calcium signaling and membrane electrophysiology (Brown and Loew, 2012) (Table 1). Future iterations of the modeling suite could additionally include the small conductance calcium-activated potassium channels (SK), which has been shown to help mediate the influence of calcium signaling on membrane electrophysiology in SCA2 and EA, and has also been proposed as a potential therapeutic targets (Alviña and Khodakhah, 2010; Kasumu et al., 2012a).

## Clinical translation

### Computational models are clinically informative for SCAs

The cerebellum is conserved across all vertebrate species (Kandel et al., 2000). Thus, a combination of computational models and mouse models is clinically informative for human SCAs. Several SCA mouse models have been developed (Burright et al., 1995; Huynh et al., 2000; van de Leemput et al., 2007; Colomer Gould, 2012; Kelp et al., 2013). As virtual model neurons are created, details of the computational models are validated by comparison with experimental data in these mice (Xu et al., 2003; Marasco et al., 2012), with an end to translation to humans (Figure 1).

### ICpeptide application *in vivo* for SCAs and other polyQ diseases

Peptides resembling portions of the IP3R1 C-terminal (ICpeptides) (Supplementary Material, Figure S1B) have been created (Tang et al., 2003b, 2009; Tu et al., 2004). SCA model results suggest that application of IC-G2736X (IP3R1 base pairs D2590-G2736) (Supplementary Material, Figure S1B) restores normal calcium transients in polyQ ataxias (Brown and Loew, 2012). ICpeptides could be used to develop more selective therapeutics that can then be tested in animals and, if promising, in patients. Simulations also showed that treating SCA15/16 mice with IC4 (IP3R1 base pairs Q2714-A2749) normalizes calcium release by therapeutically increasing IP3R1 sensitivity to IP3 to counteract IP3R1 haploinsufficiency (Brown and Loew, 2012). IC4 competitively binds protein phosphatase 1 alpha (PP1α; a phosphatase that decreases IP3R1 sensitivity).

A different polyQ disorder, Huntington's disease (HD), is sometimes phenotypically confused with SCA if ataxia is prominent (Tang et al., 2003a; Bezprozvanny and Hayden, 2004; Bezprozvanny, 2007; Zhang et al., 2008; Dong et al., 2013; Rodríguez-Quiroga et al., 2013). Whereas ataxias primarily exhibit motor discoordination, HD is a neurodegenerative hyperkinetic movement disorder affecting the basal ganglia (Bezprozvanny, 2011). Although molecular interactions in Purkinje neurons are different from those in medial striatal neurons, the polyQ SCAs share underlying pathophysiology with HD involving supersensitive IP3R1 (Bezprozvanny, 2011). Application of the IC10 peptide (IP3R1 base pairs F2627-A2749) (Supplementary Material, Figure S1B) in medial striatal neurons in HD mice restored normal calcium response (Tang et al., 2009). These mice were largely spared from neurotoxicity, with improved motor coordination (Tang et al., 2009). IC-peptides therefore represent an alternative step toward thinking about new therapeutics for polyQ disorders.

### IP3 suppression in SCAs

Subsequent to these simulations (Brown and Loew, 2012), it was demonstrated that overexpressing inositol 1,4,5-phosphatase (5PP) to chronically suppress IP3R1-mediated calcium release improved motor coordination in SCA2 mice (Kasumu et al., 2012b). The enzyme 5PP converts IP3 to the inactive form inositol 1,4-bisphosphatase (Supplementary Material, Figure S1A), decreasing the overall amount of IP3 sensed by IP3R1. This demonstrated that suppression of IP3R1-mediated calcium release could be of therapeutic benefit for SCAs, as supported by SCA modeling predictions (Figure 1).

### Anticipatory therapeutics for SCAs

There is currently no direct way of treating hereditary ataxias. Patients can be cared for symptomatically, with physical and speech therapy and walking aids (Schöls et al., 2004). If SCA modeling can help us understand how alterations in calcium signaling and membrane electrophysiology can be restored in SCAs, this can enhance our chance at engineering therapeutics for ataxia. In the era of SCA genomic testing (Smeets and Verbeek, 2014), anticipatory therapeutics would be useful preferentially in presymptomatic patients (Brown and Loew, 2015). For example, if ICpeptides are applied before mice become symptomatic, this could reduce symptom occurrence, delay onset, slow progression, and maximize output from undamaged tissue. SCA modeling would therefore be integrated with genomic testing, family history considerations, and presymptomatic investigation and treatment. Presymptomatic testing is often pursued for severe late-onset neurodegenerative diseases, including SCAs (Guimarães et al., 2013; Schuler-Faccini et al., 2014). Even without genetic testing, other early clinical disease features (prodromes) are detectable prior to onset of ataxia and can be used to determine treatment timing (Velázquez-Pérez et al., 2014a,b). Such translation, after extensive lab research, could improve quality of life and alleviate economical, social, and occupational strains on patients, as well as family members and caregivers.

### Uses for SCA modeling exemplified

In computational models, various parameters in the cerebellar Purkinje neuron may be deliberately perturbed, with results examined to see if any of these perturbations match those found in individuals with various cerebellar disorders. This can assist with making connections between subcellular dysfunction and phenotypic manifestations. In addition to understanding pathophysiology, the models could be used to screen drugs, investigate adverse effects, and examine ramifications of genetic replacements and knockouts.

There are other computational models that can be used to exemplify the potential of SCA models. Experiment-based computational modeling similar to SCA, including the use of individualized 3D reconstruction of *in vivo*-acquired computed tomography (CT) images from several patients, showed that peak wall stress calculated *in vivo* for abdominal aortic aneurysm (AAA) near the time of rupture was more predictive of rupture than the conventional assessment of aortic diameter (Fillinger et al., 2002; Raut et al., 2013; Soudah et al., 2013). Similarly, Caroli et al used computational modeling to create patient-specific computational vascular network models (Caroli et al., 2013). These models predicted blood flow 6 weeks after surgical creation of arteriovenous fistulas (AVF) for long-term hemodialysis (Caroli et al., 2013). The model was validated in a multicenter, prospective clinical study, and is expected to reduce AVF failure or dysfunction and related patient morbidity. A line of computational models were also created to compare various modes of pharmacologic delivery of Doxorubicin, a chemotherapeutic drug used for a variety of cancers. The models predicted optimal antitumor efficacy with protection from adverse effects with thermosensitive liposomes or with an administration protocol using increased duration of infusion with higher doses, compared to the conventional bolus injection (Reich et al., 1979; El-Kareh and Secomb, 2000, 2005; Evans et al., 2009; Zhan and Xu, 2013).

### Systems biology in translational medicine

A number of scientific contributions from computational modeling (Table 1) that enhance our understanding of SCA have been presented. The computational models are more economical, reproducible, and expedient than animal experiments. However, the computer models depend on and integrate information from animal studies and clinical observations. As a result, Figure 1 shows that animal experiments and SCA models work together in concert to iteratively explain and predict pathophysiology. In addition, computational predictions were made via a bioinformatics screen to identify transcripts that interact with and have functions relevant to polyQ SCAs (Spence and Wallihan, 2012). Next steps in translational systems biology will include mining and modeling network motifs in ataxia and considering their functional implications (Alon, 2007; Brown and Loew, 2015). Findings from such translational studies could be used to develop new drugs or treatment strategies (Matilla-Dueñas et al., 2014), suggesting that computational neurobiology plays a role in translational neurology (Figure 1). Expanding interactions between these related research and medical communities (De Schutter, 2008) will usher in an era that may create advanced support for Translational Medicine and, in particular, Neurology.

## Author contributions

Sherry-Ann Brown conceived of, analyzed, designed, drafted, critically revised, approved, and agreed to be accountable for this submitted work. Louise D. McCullough conceived of, critically revised, approved, and agreed to be accountable for this submitted work. Leslie M. Loew analyzed, critically revised, approved, and agreed to be accountable for this submitted work.

### Conflict of interest statement

The authors declare that the research was conducted in the absence of any commercial or financial relationships that could be construed as a potential conflict of interest.
