# Supplementary material for: Computational neurobiology is a useful tool in translational neurology: the example of ataxia
Source: Front Neurosci. 2015 Jan 21;9:1. doi: 10.3389/fnins.2015.00001 (PMC4300942; doi:10.3389/fnins.2015.00001)
Supplement: Supplementary file 1 [file DataSheet1.DOCX]

**Supplementary Material**

**Computational neurobiology is a useful tool in translational neurology: the example of ataxia**

**Sherry-Ann Brown^1*^, Louise D. McCullough LD^2^, Leslie M. Loew^3^**

^1^ Department of Medicine, Mayo Clinic, Rochester, MN, USA

^2^ Department of Neurology, University of Connecticut Health Center, Farmington, CT, USA

^3^ Richard D. Berlin Center for Cell Analysis & Modeling, University of Connecticut Health Center, Farmington, CT, USA

**^*^ Correspondence:** Dr. Sherry-Ann Brown, Mayo Clinic, 200 First Street SW, Rochester, MN, 55905, USA.

brown.sherryann@mayo.edu

1. **Supplementary Data**

**S1 Signaling complex regulation**

**S1.1 Downregulation of signaling complex proteins in SCA**

The concerted reduction in gene expression of related calcium signaling proteins raises the possibility of what could be termed ‘signaling complex regulation’. Several studies suggest that glutamatergic signaling proteins coimmunoprecipitate, and may exist at the plasma membrane in the form of complexes (Fig. S1b and Table S1) (Tu, Xiao et al. 1998, Hirota, Ando et al. 2003, Sandonà, Scolari et al. 2003, Nakamura, Sato et al. 2004, Mikoshiba 2007). Various groups propose that plasma membrane calcium ATP-ase (PMCA), mGluR, Homer, and IP3R1 form a multimeric calcium signaling complex (Tu, Xiao et al. 1998, Sandonà, Scolari et al. 2003, Nakamura, Sato et al. 2004, Kurnellas, Lee et al. 2007). One study also suggests that protein kinase A (PKA), protein phosphatase 1 (PP1), and protein phosphatase 2A (PP2A) may be part of this complex with IP3R1 (DeSouza, Reiken et al. 2002). This is supported by the cooperative findings that mGluR, glutamate receptor δ2 (Grid2), excitatory amino-acid transporter type 4 (EAAT4; a glutamate transporter), phospholipase C (PLC, enzyme activated by mGluR activity), IP3R1 (calcium channel which senses more IP3 in the presence of mGluR activity), plasma membrane calcium ATPase pump (PMCA), Homer, and other molecules are reduced in various combinations in several ataxias (Lin, Antalffy et al. 2000, Serra, Byam et al. 2004, Chou, Yeh et al. 2008, Kato, Knierman et al. 2012), likely with common transcription factors (such as retinoid acid receptor-related orphan receptor alpha (RORα) (Fig. S1b) (Serra, Duvick et al. 2006, Gehrking, Andresen et al. 2011, Euler, Friedrich et al. 2012)).

**S1.2 Nuclear localization of mutant ataxin proteins downregulates transcription**

While Ataxin1 is found in the nucleus, Ataxin 2 and Ataxin3 are cytoplasmic proteins, under normal conditions. However, in brains from patients with SCA1 and SCA3, the mutant protein also accumulates in the nucleus (Fig. S1b) (Servadio, Koshy et al. 1995, Paulson, Perez et al. 1997, Klement, Skinner et al. 1998, Schmidt, Lindenberg et al. 2002). The mutant Ataxin-2 and Ataxin-3 proteins directly operate in the nucleus to downregulate transcription of these calcium signaling genes (Lin, Antalffy et al. 2000, Chou, Yeh et al. 2008). Experiment-based simulations explain the impact of signaling complex downregulation in SCA, and suggest that this leads to partial compensation for hyperactive IP3R1-mediated calcium release (Brown and Loew 2012). The signaling complex regulation hypothesis is supported by the close proximity of sER to the plasma membrane, so that IP3R1 on the surface of the sER is a component of submembrane signaling complexes (Sandonà, Scolari et al. 2003). A hypothesis for the latter is further supported by the suggestion that some smooth ER is located close to the plasma membrane, so that the IP3R1 on the surface of the smooth ER is a component of submembrane signaling complexes (Sandonà, Scolari et al. 2003). In fact, de Graaf et al found that various groups of proteins that are normally specifically expressed and active in Purkinje pre- and post-synaptic domains were jointly downregulated in the cerebellum of aging neurodegenerative mice, including Homer scaffolding proteins, mGluR neurotransmitter receptors, various ion channels/transporters (including SERCA3), and signal transduction enzymes (e.g., PKC) (de Graaf, Vermeij et al. 2013). It has thus been proposed that these submembrane clusters (Fig. S1b and Table S1) may ‘define spatially localized signaling’ (Sandonà, Scolari et al. 2003) and optimize calcium release and signaling.

**S1.3 Signaling complexes localize molecules without restricting diffusion**

Homer (3, 1b/c, and so on) is a family of postsynaptic scaffolding proteins that form components of the submembrane signaling complexes (Fig. S1b). Homer is therefore involved in calcium signaling at the plasma membrane-smooth endoplasmic reticulum (sER) interface (Tu, Xiao et al. 1998, Sandonà, Scolari et al. 2003). It is unclear whether Homer physically tethers IP3R1 (Tu, Xiao et al. 1998) in the sER at the postsynaptic density (PSD) to functionally link glutamatergic signaling to IP3R1-related calcium release. Analysis of electron microscope images from (Xiao, Tu et al. 1998) suggest that Homer is as much as 200nm from the plasma membrane, which correlates with similar distances observed for sER (Harris and Stevens 1988, Xu-Friedman, Harris et al. 2001). Based on SCA modeling results, this distance is not sufficient to compartmentalize or restrict diffusion of molecules such as calcium and IP3 which have diffusion coefficients of 223 µm^2^.s^-1^ and 283 µm^2^.s^-1^, respectively (Allbritton, Meyer et al. 1992). Thus, the proximity of IP3R1 on the sER to Homer beneath the plasma membrane and mGluR on the plasma membrane may allow for localization of cellular processes without restriction of diffusion.

**Supplementary Figures and Tables**

## Supplementary Tables

**Supplemental Table S1 Ataxias in humans and mice involving a variety of calcium signaling proteins**

|  |  |  |
| --- | --- | --- |
| **Disorder** | **Mutant protein** | **References** |
| Smooth endoplasmic reticulum cluster |  |  |
| Spinocerebellar Ataxia 1 (SCA1) | Ataxin 1 | (Orr, Chung et al. 1993, Burright, Clark et al. 1995) |
| Spinocerebellar Ataxia 2 (SCA2) | Ataxin 2 | (Pulst, Nechiporuk et al. 1996) |
| Spinocerebellar Ataxia 3 (SCA3) | Ataxin 3 | (Dürr, Stevanin et al. 1996, Paulson, Perez et al. 1997) |
| Spinocerebellar Ataxia 7 (SCA7)  Spinocerebellar Ataxia 8 (SCA8) | Ataxin 7  Ataxin 8 | (David, Abbas et al. 1997)  (Moseley, Zu et al. 2006) |
| Spinocerebellar Ataxia 17 (SCA17) | Ataxin 17 | (Nakamura, Jeong et al. 2001) |
| Huntington’s Disease (HD) | Huntingtin | (Datta, Choudhury et al. 2011, Euler, Friedrich et al. 2012) |
| Dentatorubral-pallidoluysian atrophy (with ataxia)(DLPRA) | DLPRA | (Liu, Tang et al. 2009, Suzuki, Zhou et al. 2012) |
| Spinocerebellar Ataxia 15 (SCA15) | IP3R1 | (van de Leemput, Chandran et al. 2007, Hara, Shiga et al. 2008, Di Gregorio, Orsi et al. 2010) |
| Spinocerebellar Ataxia 16 (SCA16) | IP3R1 | (Iwaki, Kawano et al. 2008) |
| Quadrupedal Gait ataxia (QG) | CA8 | (Türkmen, Guo et al. 2009) |
| Plasma membrane cluster |  |  |
| Ataxia/hearing loss in mice, hearing loss in humans (A-HL) | PMCA | (Kurnellas, Lee et al. 2007) |
| Autosomal-recessive congenital cerebellar ataxia (ARCCA) | mGluR | (Guergueltcheva, Azmanov et al. 2012) |
| Episodic Ataxia 2 (EA2/leaner) | Cav2.1 | (Guida, Trettel et al. 2001, Murchison, Dove et al. 2002, Mantuano, Veneziano et al. 2004, Tonelli, D'Angelo et al. 2006) |
| Spinocerebellar Ataxia 6 (SCA6) | Cav2.1 | (Ishikawa, Tanaka et al. 1997) |
| Spinocerebellar Ataxia 14 (SCA14) | PKC-γ | (Alonso, Costa et al. 2005, van Gaalen, Vermeer et al. 2013, Ji, Hassler et al. 2014) |
| Spinocerebellar Ataxia 5 (SCA5) | Spectrin β | (Ikeda, Dick et al. 2006) |
| Episodic Ataxia 6 (EA6) | EAAT1 | (de Vries, Mamsa et al. 2009) |
| Episodic Ataxia 5 (EA5) | CavB4 | (Escayg, De Waard et al. 2000) |
| Episodic Ataxia 1 (EA1)  Spinocerebellar Ataxia 13 (SCA13)  Spinocerebellar Ataxia 19 (SCA19)  Spinocerebellar Ataxia 22 (SCA22) | Kv1.1  Kv3.3  Kv4.3  Kv4.3 | (Imbrici, D'Adamo et al. 2006)  (Waters, Minassian et al. 2006)  (Duarri, Jezierska et al. 2012)  (Lee, Durr et al. 2012) |

IP3R1, IP3-gated calcium channel on the smooth endoplasmic reticulum (sER); PKC-γ, protein kinase expressed in Purkinje neurons and activated by calcium and diacylglycerol (DAG); DAG, a product of hydrolysis of phosphatidylinositol 4,5-bisphosphate (PIP2) by phospholipase C (PLC); PIP2, a phospholipid on the inner leaflet of the plasma membrane; PLC, an enzyme that hydrolyzes PIP2 to give rise to DAG and IP3 when activated by G-betagamma from metabotropic glutamate receptor type 1 (mGluR or Grm1); CA8, Carbonic anhydrase 8, an IP3R1 antagonist (Türkmen, Guo et al. 2009); EAAT1, Excitatory amino-acid transporter type 1, a glutamate transporter on the plasma membrane; Spectrin β, a plasma membrane anchor for excitatory amino-acid transporter type 1 (EAAT4; glutamate transporter) and GluRδ2 (Grid2, glutamate receptor); PMCA, Plasma membrane calcium ATP-ase, an active transporter of calcium out of the cell; Cav2.1, the main P-type calcium channel in Purkinje neurons, with nonsense/missense mutations causing episodic ataxia type 2 and expansion of CAG repeats causing spinocerebellar ataxia 6; CavB4, an accessory subunit that regulates the P-type channels encoded by Cav2.1; Kv1.1, Kv3.3, and Kv4.3, potassium channels that contribute to repolarization of the Purkinje neuron dendrite calcium spikes. Adapted from (Schorge, van de Leemput et al. 2010).

## Supplementary Figures

**Supplemental Figure S1 Calcium signaling in neurobiology**. ***(A)***, PF, parallel fibers that activate Purkinje neuron spines; CF, climbing fibers that stimulate Purkinje neuron somata and dendrites; K Ch, potassium channels including Kv1.1 (mutated in Episodic Ataxia type 1(Imbrici, Cusimano et al. 2003)), Kv3.3 (mutated in SCA13 (Waters, Minassian et al. 2006)), Kv4.3 (mutated in SCA19 (Duarri, Jezierska et al. 2012)and SCA22(Lee, Durr et al. 2012)), D-type potassium channels, BK, and SK, which are respectively, four potassium channels involved in Purkinje membrane repolarization, the large conductance calcium-activated voltage-gated potassium channel, and the small conductance calcium-activated voltage-independent potassium channel; DAG, diacylglycerol a product of PLC hydrolysis that activates PKC along with calcium; PIP2, Phosphatidylinositol 4,5-bisphosphate, a plasma membrane phospholipid of the inner leaflet that gives rise to DAG and IP3 when hydrolyzed; PLC, phospholipase C, an enzyme that hydrolyzes PIP2 when activated by G-βγ from mGluR; mGluR or Grm1, metabotropic glutamate receptor type 1 (Guergueltcheva, Azmanov et al. 2012); B, calcium-binding proteins or buffers including calbindin and parvalbumin; SERCA, sarcoendoplasmic reticulum calcium ATPase, a transporter for calcium entry from the cytosol to the smooth endoplasmic reticulum (sER); RYR, ryanodine receptor, a transporter from calcium exit from the ER to the cytosol; Ca Ch, calcium channels including class-E calcium channels and Cav2.1 (Cav2.1 is the main P-type calcium channel in PCs with nonsense/missense mutations causing episodic ataxia type 2 and expansion of CAG repeats causing SCA6, while mutations in CavB4 an accessory subunit for Cav2.1 cause EA5 (Escayg, De Waard et al. 2000)); IP3R, inositol trisphosphate receptor (mutated in SCA15/16 and altered sensitivity in SCA1-3), intracellular calcium release channel on the sER gated by IP3; PKC, protein kinase C (mutated in SCA14) expressed in Purkinje neurons that helps control expression of surface molecules. Adapted from (Hernjak, Slepchenko et al. 2005). (***B)***, Signaling complex formation in the Purkinje spiny dendrite including additional molecules such as Excitatory amino-acid transporter type 4 (EAAT4; a glutamate transporter), Glutamate receptor δ2 (Grid2), Homer 3 and Homer 1 b/c (scaffolding proteins), Myosin Va (which along with Homer pulls sER into the spine head); IC (ICpeptides – IC-10, IC-4, IC-G2736X) in the cytosol, mAtaxin (mutant Ataxin proteins) in the cytosol and nucleus, and the transcription factor retinoid acid receptor-related orphan receptor alpha (RORα) in the nucleus are also present nearby as shown.

1. **References**

Allbritton, N., T. Meyer and L. Stryer (1992). "Range of messenger action of calcium ion and inositol 1,4,5-trisphosphate." Science **258**(5089): 1812-1815.

Alonso, I., C. Costa, A. Gomes, A. Ferro, A. Seixas, S. Silva, V. Cruz, P. Coutinho, J. Sequeiros and I. Silveira (2005). "A novel H101Q mutation causes PKCgamma loss in spinocerebellar ataxia type 14." J Hum Genet **50**(10): 523-529.

Brown, S. A. and L. M. Loew (2012). "Computational analysis of calcium signaling and membrane electrophysiology in cerebellar Purkinje neurons associated with ataxia." BMC Syst Biol **6**(1): 70.

Burright, E., H. Clark, A. Servadio, T. Matilla, R. Feddersen, W. Yunis, L. Duvick, H. Zoghbi and H. Orr (1995). "SCA1 transgenic mice: a model for neurodegeneration caused by an expanded CAG trinucleotide repeat." Cell **82**(6): 937-948.

Chou, A., T. Yeh, P. Ouyang, Y. Chen, S. Chen and H. Wang (2008). "Polyglutamine-expanded ataxin-3 causes cerebellar dysfunction of SCA3 transgenic mice by inducing transcriptional dysregulation." Neurobiol Dis **31**(1): 89-101.

Datta, M., A. Choudhury, A. Lahiri and N. P. Bhattacharyya (2011). "Genome wide gene expression regulation by HIP1 Protein Interactor, HIPPI: prediction and validation." BMC Genomics **12**: 463.

David, G., N. Abbas, G. Stevanin, A. Dürr, G. Yvert, G. Cancel, C. Weber, G. Imbert, F. Saudou, E. Antoniou, H. Drabkin, R. Gemmill, P. Giunti, A. Benomar, N. Wood, M. Ruberg, Y. Agid, J. Mandel and A. Brice (1997). "Cloning of the SCA7 gene reveals a highly unstable CAG repeat expansion." Nat Genet **17**(1): 65-70.

de Graaf, E. L., W. P. Vermeij, M. C. de Waard, Y. Rijksen, I. van der Pluijm, C. C. Hoogenraad, J. H. Hoeijmakers, A. F. Altelaar and A. J. Heck (2013). "Spatio-temporal analysis of molecular determinants of neuronal degeneration in the aging mouse cerebellum." Mol Cell Proteomics **12**(5): 1350-1362.

de Vries, B., H. Mamsa, A. H. Stam, J. Wan, S. L. Bakker, K. R. Vanmolkot, J. Haan, G. M. Terwindt, E. M. Boon, B. D. Howard, R. R. Frants, R. W. Baloh, M. D. Ferrari, J. C. Jen and A. M. van den Maagdenberg (2009). "Episodic ataxia associated with EAAT1 mutation C186S affecting glutamate reuptake." Arch Neurol **66**(1): 97-101.

DeSouza, N., S. Reiken, K. Ondrias, Y. Yang, S. Matkovich and A. Marks (2002). "Protein kinase A and two phosphatases are components of the inositol 1,4,5-trisphosphate receptor macromolecular signaling complex." J Biol Chem **277**(42): 39397-39400.

Di Gregorio, E., L. Orsi, M. Godani, G. Vaula, S. Jensen, E. Salmon, G. Ferrari, S. Squadrone, M. Abete, C. Cagnoli, A. Brussino and A. Brusco (2010). "Two Italian Families with ITPR1 Gene Deletion Presenting a Broader Phenotype of SCA15." Cerebellum.

Duarri, A., J. Jezierska, M. Fokkens, M. Meijer, H. J. Schelhaas, W. F. den Dunnen, F. van Dijk, C. Verschuuren-Bemelmans, G. Hageman, P. van de Vlies, B. Küsters, B. P. van de Warrenburg, B. Kremer, C. Wijmenga, R. J. Sinke, M. A. Swertz, H. H. Kampinga, E. Boddeke and D. S. Verbeek (2012). "Mutations in potassium channel kcnd3 cause spinocerebellar ataxia type 19." Ann Neurol **72**(6): 870-880.

Dürr, A., G. Stevanin, G. Cancel, C. Duyckaerts, N. Abbas, O. Didierjean, H. Chneiweiss, A. Benomar, O. Lyon-Caen, J. Julien, M. Serdaru, C. Penet, Y. Agid and A. Brice (1996). "Spinocerebellar ataxia 3 and Machado-Joseph disease: clinical, molecular, and neuropathological features." Ann Neurol **39**(4): 490-499.

Escayg, A., M. De Waard, D. D. Lee, D. Bichet, P. Wolf, T. Mayer, J. Johnston, R. Baloh, T. Sander and M. H. Meisler (2000). "Coding and noncoding variation of the human calcium-channel beta4-subunit gene CACNB4 in patients with idiopathic generalized epilepsy and episodic ataxia." Am J Hum Genet **66**(5): 1531-1539.

Euler, P., B. Friedrich, R. Ziegler, A. Kuhn, K. S. Lindenberg, C. Weiller and B. Zucker (2012). "Gene expression analysis on a single cell level in Purkinje cells of Huntington's disease transgenic mice." Neurosci Lett **517**(1): 7-12.

Gehrking, K. M., J. M. Andresen, L. Duvick, J. Lough, H. Y. Zoghbi and H. T. Orr (2011). "Partial loss of Tip60 slows mid-stage neurodegeneration in a spinocerebellar ataxia type 1 (SCA1) mouse model." Hum Mol Genet **20**(11): 2204-2212.

Guergueltcheva, V., D. N. Azmanov, D. Angelicheva, K. R. Smith, T. Chamova, L. Florez, M. Bynevelt, T. Nguyen, S. Cherninkova, V. Bojinova, A. Kaprelyan, L. Angelova, B. Morar, D. Chandler, R. Kaneva, M. Bahlo, I. Tournev and L. Kalaydjieva (2012). "Autosomal-recessive congenital cerebellar ataxia is caused by mutations in metabotropic glutamate receptor 1." Am J Hum Genet **91**(3): 553-564.

Guida, S., F. Trettel, S. Pagnutti, E. Mantuano, A. Tottene, L. Veneziano, T. Fellin, M. Spadaro, K. Stauderman, M. Williams, S. Volsen, R. Ophoff, R. Frants, C. Jodice, M. Frontali and D. Pietrobon (2001). "Complete loss of P/Q calcium channel activity caused by a CACNA1A missense mutation carried by patients with episodic ataxia type 2." Am J Hum Genet **68**(3): 759-764.

Hara, K., A. Shiga, H. Nozaki, J. Mitsui, Y. Takahashi, H. Ishiguro, H. Yomono, H. Kurisaki, J. Goto, T. Ikeuchi, S. Tsuji, M. Nishizawa and O. Onodera (2008). "Total deletion and a missense mutation of ITPR1 in Japanese SCA15 families." Neurology **71**(8): 547-551.

Harris, K. and J. Stevens (1988). "Dendritic spines of rat cerebellar Purkinje cells: serial electron microscopy with reference to their biophysical characteristics." J Neurosci **8**(12): 4455-4469.

Hernjak, N., B. Slepchenko, K. Fernald, C. Fink, D. Fortin, I. Moraru, J. Watras and L. Loew (2005). "Modeling and analysis of calcium signaling events leading to long-term depression in cerebellar Purkinje cells." Biophys J **89**(6): 3790-3806.

Hirota, J., H. Ando, K. Hamada and K. Mikoshiba (2003). "Carbonic anhydrase-related protein is a novel binding protein for inositol 1,4,5-trisphosphate receptor type 1." Biochem J **372**(Pt 2): 435-441.

Ikeda, Y., K. A. Dick, M. R. Weatherspoon, D. Gincel, K. R. Armbrust, J. C. Dalton, G. Stevanin, A. Dürr, C. Zühlke, K. Bürk, H. B. Clark, A. Brice, J. D. Rothstein, L. J. Schut, J. W. Day and L. P. Ranum (2006). "Spectrin mutations cause spinocerebellar ataxia type 5." Nat Genet **38**(2): 184-190.

Imbrici, P., A. Cusimano, M. D'Adamo, A. De Curtis and M. Pessia (2003). "Functional characterization of an episodic ataxia type-1 mutation occurring in the S1 segment of hKv1.1 channels." Pflugers Arch **446**(3): 373-379.

Imbrici, P., M. D'Adamo, D. Kullmann and M. Pessia (2006). "Episodic ataxia type 1 mutations in the KCNA1 gene impair the fast inactivation properties of the human potassium channels Kv1.4-1.1/Kvbeta1.1 and Kv1.4-1.1/Kvbeta1.2." Eur J Neurosci **24**(11): 3073-3083.

Ishikawa, K., H. Tanaka, M. Saito, N. Ohkoshi, T. Fujita, K. Yoshizawa, T. Ikeuchi, M. Watanabe, A. Hayashi, Y. Takiyama, M. Nishizawa, I. Nakano, K. Matsubayashi, M. Miwa, S. Shoji, I. Kanazawa, S. Tsuji and H. Mizusawa (1997). "Japanese families with autosomal dominant pure cerebellar ataxia map to chromosome 19p13.1-p13.2 and are strongly associated with mild CAG expansions in the spinocerebellar ataxia type 6 gene in chromosome 19p13.1." Am J Hum Genet **61**(2): 336-346.

Iwaki, A., Y. Kawano, S. Miura, H. Shibata, D. Matsuse, W. Li, H. Furuya, Y. Ohyagi, T. Taniwaki, J. Kira and Y. Fukumaki (2008). "Heterozygous deletion of ITPR1, but not SUMF1, in spinocerebellar ataxia type 16." J Med Genet **45**(1): 32-35.

Ji, J., M. L. Hassler, E. Shimobayashi, N. Paka, R. Streit and J. P. Kapfhammer (2014). "Increased protein kinase C gamma activity induces Purkinje cell pathology in a mouse model of spinocerebellar ataxia 14." Neurobiol Dis **70C**: 1-11.

Kato, A. S., M. D. Knierman, E. R. Siuda, J. T. Isaac, E. S. Nisenbaum and D. S. Bredt (2012). "Glutamate receptor δ2 associates with metabotropic glutamate receptor 1 (mGluR1), protein kinase Cγ, and canonical transient receptor potential 3 and regulates mGluR1-mediated synaptic transmission in cerebellar Purkinje neurons." J Neurosci **32**(44): 15296-15308.

Klement, I., P. Skinner, M. Kaytor, H. Yi, S. Hersch, H. Clark, H. Zoghbi and H. Orr (1998). "Ataxin-1 nuclear localization and aggregation: role in polyglutamine-induced disease in SCA1 transgenic mice." Cell **95**(1): 41-53.

Kurnellas, M., A. Lee, H. Li, L. Deng, D. Ehrlich and S. Elkabes (2007). "Molecular alterations in the cerebellum of the plasma membrane calcium ATPase 2 (PMCA2)-null mouse indicate abnormalities in Purkinje neurons." Mol Cell Neurosci **34**(2): 178-188.

Lee, Y. C., A. Durr, K. Majczenko, Y. H. Huang, Y. C. Liu, C. C. Lien, P. C. Tsai, Y. Ichikawa, J. Goto, M. L. Monin, J. Z. Li, M. Y. Chung, E. Mundwiller, V. Shakkottai, T. T. Liu, C. Tesson, Y. C. Lu, A. Brice, S. Tsuji, M. Burmeister, G. Stevanin and B. W. Soong (2012). "Mutations in KCND3 cause spinocerebellar ataxia type 22." Ann Neurol **72**(6): 859-869.

Lin, X., B. Antalffy, D. Kang, H. Orr and H. Zoghbi (2000). "Polyglutamine expansion down-regulates specific neuronal genes before pathologic changes in SCA1." Nat Neurosci **3**(2): 157-163.

Liu, J., T. Tang, H. Tu, O. Nelson, E. Herndon, D. Huynh, S. Pulst and I. Bezprozvanny (2009). "Deranged calcium signaling and neurodegeneration in spinocerebellar ataxia type 2." J Neurosci **29**(29): 9148-9162.

Mantuano, E., L. Veneziano, M. Spadaro, P. Giunti, S. Guida, M. Leggio, L. Verriello, N. Wood, C. Jodice and M. Frontali (2004). "Clusters of non-truncating mutations of P/Q type Ca2+ channel subunit Ca(v)2.1 causing episodic ataxia 2." J Med Genet **41**(6): e82.

Mikoshiba, K. (2007). "IP3 receptor/Ca2+ channel: from discovery to new signaling concepts." Journal of neurochemistry **102**(5): 1426-1446.

Moseley, M. L., T. Zu, Y. Ikeda, W. Gao, A. K. Mosemiller, R. S. Daughters, G. Chen, M. R. Weatherspoon, H. B. Clark, T. J. Ebner, J. W. Day and L. P. Ranum (2006). "Bidirectional expression of CUG and CAG expansion transcripts and intranuclear polyglutamine inclusions in spinocerebellar ataxia type 8." Nat Genet **38**(7): 758-769.

Murchison, D., L. Dove, L. Abbott and W. Griffith (2002). "Homeostatic compensation maintains Ca2+ signaling functions in Purkinje neurons in the leaner mutant mouse." Cerebellum **1**(2): 119-127.

Nakamura, K., S. Jeong, T. Uchihara, M. Anno, K. Nagashima, T. Nagashima, S. Ikeda, S. Tsuji and I. Kanazawa (2001). "SCA17, a novel autosomal dominant cerebellar ataxia caused by an expanded polyglutamine in TATA-binding protein." Hum Mol Genet **10**(14): 1441-1448.

Nakamura, M., K. Sato, M. Fukaya, K. Araishi, A. Aiba, M. Kano and M. Watanabe (2004). "Signaling complex formation of phospholipase Cbeta4 with metabotropic glutamate receptor type 1alpha and 1,4,5-trisphosphate receptor at the perisynapse and endoplasmic reticulum in the mouse brain." Eur J Neurosci **20**(11): 2929-2944.

Orr, H., M. Chung, S. Banfi, T. J. Kwiatkowski, A. Servadio, A. Beaudet, A. McCall, L. Duvick, L. Ranum and H. Zoghbi (1993). "Expansion of an unstable trinucleotide CAG repeat in spinocerebellar ataxia type 1." Nat Genet **4**(3): 221-226.

Paulson, H., M. Perez, Y. Trottier, J. Trojanowski, S. Subramony, S. Das, P. Vig, J. Mandel, K. Fischbeck and R. Pittman (1997). "Intranuclear inclusions of expanded polyglutamine protein in spinocerebellar ataxia type 3." Neuron **19**(2): 333-344.

Pulst, S., A. Nechiporuk, T. Nechiporuk, S. Gispert, X. Chen, I. Lopes-Cendes, S. Pearlman, S. Starkman, G. Orozco-Diaz, A. Lunkes, P. DeJong, G. Rouleau, G. Auburger, J. Korenberg, C. Figueroa and S. Sahba (1996). "Moderate expansion of a normally biallelic trinucleotide repeat in spinocerebellar ataxia type 2." Nat Genet **14**(3): 269-276.

Sandonà, D., A. Scolari, K. Mikoshiba and P. Volpe (2003). "Subcellular distribution of Homer 1b/c in relation to endoplasmic reticulum and plasma membrane proteins in Purkinje neurons." Neurochem Res **28**(8): 1151-1158.

Schmidt, T., K. Lindenberg, A. Krebs, L. Schöls, F. Laccone, J. Herms, M. Rechsteiner, O. Riess and G. Landwehrmeyer (2002). "Protein surveillance machinery in brains with spinocerebellar ataxia type 3: redistribution and differential recruitment of 26S proteasome subunits and chaperones to neuronal intranuclear inclusions." Ann Neurol **51**(3): 302-310.

Schorge, S., J. van de Leemput, A. Singleton, H. Houlden and J. Hardy (2010). "Human ataxias: a genetic dissection of inositol triphosphate receptor (ITPR1)-dependent signaling." Trends Neurosci.

Serra, H., C. Byam, J. Lande, S. Tousey, H. Zoghbi and H. Orr (2004). "Gene profiling links SCA1 pathophysiology to glutamate signaling in Purkinje cells of transgenic mice." Hum Mol Genet **13**(20): 2535-2543.

Serra, H., L. Duvick, T. Zu, K. Carlson, S. Stevens, N. Jorgensen, A. Lysholm, E. Burright, H. Zoghbi, H. Clark, J. Andresen and H. Orr (2006). "RORalpha-mediated Purkinje cell development determines disease severity in adult SCA1 mice." Cell **127**(4): 697-708.

Servadio, A., B. Koshy, D. Armstrong, B. Antalffy, H. Orr and H. Zoghbi (1995). "Expression analysis of the ataxin-1 protein in tissues from normal and spinocerebellar ataxia type 1 individuals." Nat Genet **10**(1): 94-98.

Suzuki, K., J. Zhou, T. Sato, K. Takao, T. Miyagawa, M. Oyake, M. Yamada, H. Takahashi, Y. Takahashi, J. Goto and S. Tsuji (2012). "DRPLA transgenic mouse substrains carrying single copy of full-length mutant human DRPLA gene with variable sizes of expanded CAG repeats exhibit CAG repeat length- and age-dependent changes in behavioral abnormalities and gene expression profiles." Neurobiol Dis **46**(2): 336-350.

Tonelli, A., M. D'Angelo, R. Salati, L. Villa, C. Germinasi, T. Frattini, G. Meola, A. Turconi, N. Bresolin and M. Bassi (2006). "Early onset, non fluctuating spinocerebellar ataxia and a novel missense mutation in CACNA1A gene." J Neurol Sci **241**(1-2): 13-17.

Tu, J., B. Xiao, J. Yuan, A. Lanahan, K. Leoffert, M. Li, D. Linden and P. Worley (1998). "Homer binds a novel proline-rich motif and links group 1 metabotropic glutamate receptors with IP3 receptors." Neuron **21**(4): 717-726.

Türkmen, S., G. Guo, M. Garshasbi, K. Hoffmann, A. J. Alshalah, C. Mischung, A. Kuss, N. Humphrey, S. Mundlos and P. N. Robinson (2009). "CA8 mutations cause a novel syndrome characterized by ataxia and mild mental retardation with predisposition to quadrupedal gait." PLoS Genet **5**(5): e1000487.

van de Leemput, J., J. Chandran, M. Knight, L. Holtzclaw, S. Scholz, M. Cookson, H. Houlden, K. Gwinn-Hardy, H. Fung, X. Lin, D. Hernandez, J. Simon-Sanchez, N. Wood, P. Giunti, I. Rafferty, J. Hardy, E. Storey, R. Gardner, S. Forrest, E. Fisher, J. Russell, H. Cai and A. Singleton (2007). "Deletion at ITPR1 underlies ataxia in mice and spinocerebellar ataxia 15 in humans." PLoS Genet **3**(6): e108.

van Gaalen, J., S. Vermeer, M. van Veluw, B. P. van de Warrenburg and D. Dooijes (2013). "A de novo SCA14 mutation in an isolated case of late-onset cerebellar ataxia." Mov Disord **28**(13): 1902-1903.

Waters, M. F., N. A. Minassian, G. Stevanin, K. P. Figueroa, J. P. Bannister, D. Nolte, A. F. Mock, V. G. Evidente, D. B. Fee, U. Müller, A. Dürr, A. Brice, D. M. Papazian and S. M. Pulst (2006). "Mutations in voltage-gated potassium channel KCNC3 cause degenerative and developmental central nervous system phenotypes." Nat Genet **38**(4): 447-451.

Xiao, B., J. Tu, R. Petralia, J. Yuan, A. Doan, C. Breder, A. Ruggiero, A. Lanahan, R. Wenthold and P. Worley (1998). "Homer regulates the association of group 1 metabotropic glutamate receptors with multivalent complexes of homer-related, synaptic proteins." Neuron **21**(4): 707-716.

Xu-Friedman, M., K. Harris and W. Regehr (2001). "Three-dimensional comparison of ultrastructural characteristics at depressing and facilitating synapses onto cerebellar Purkinje cells." J Neurosci **21**(17): 6666-6672.
