# Supplementary figures and images for: Computational neurobiology is a useful tool in translational neurology: the example of ataxia
Source: Front Neurosci. 2015 Jan 21;9:1. doi: 10.3389/fnins.2015.00001 (PMC4300942; doi:10.3389/fnins.2015.00001)

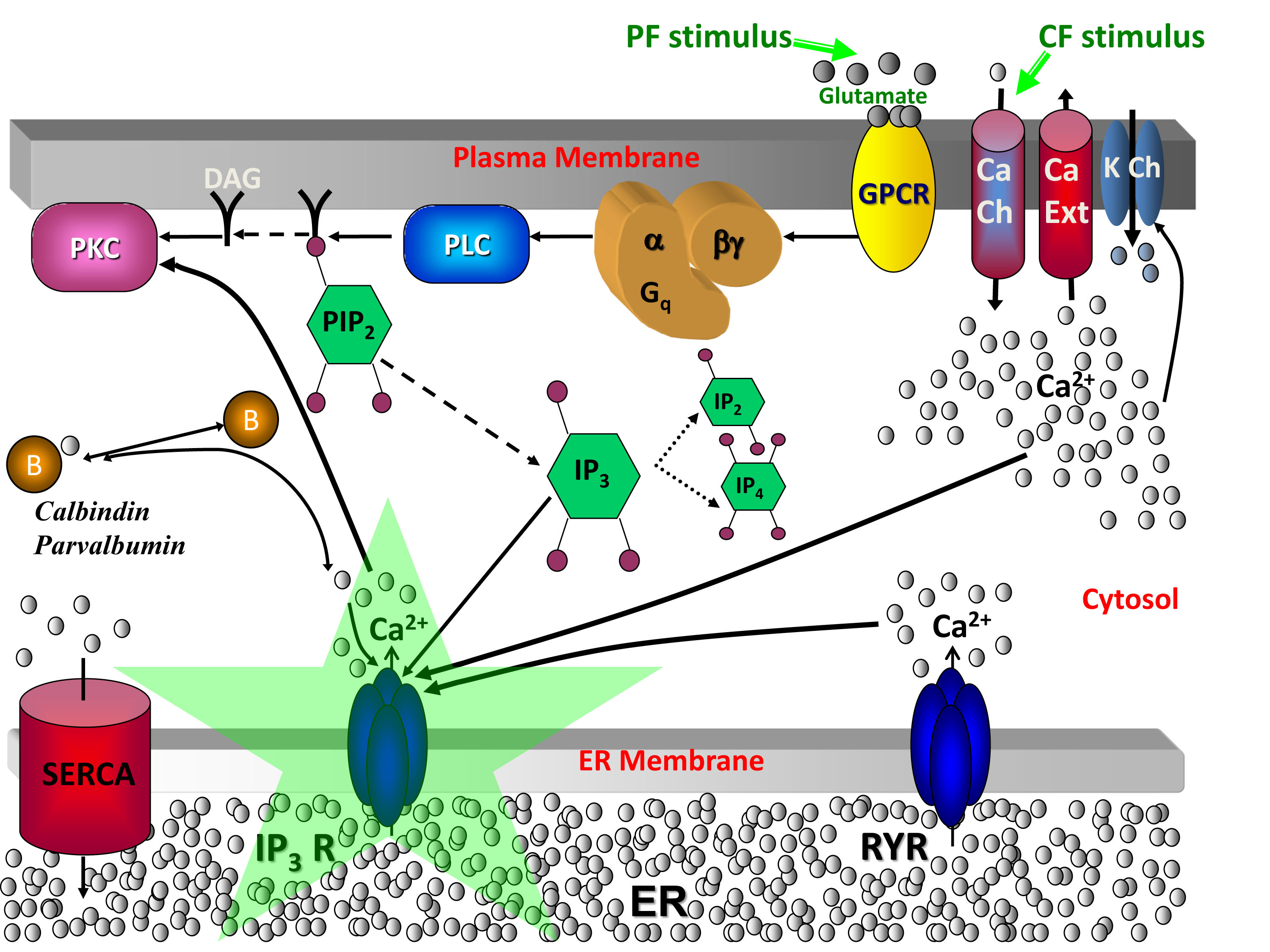

Supplement: Supplementary file 2 [file Image1.TIF]

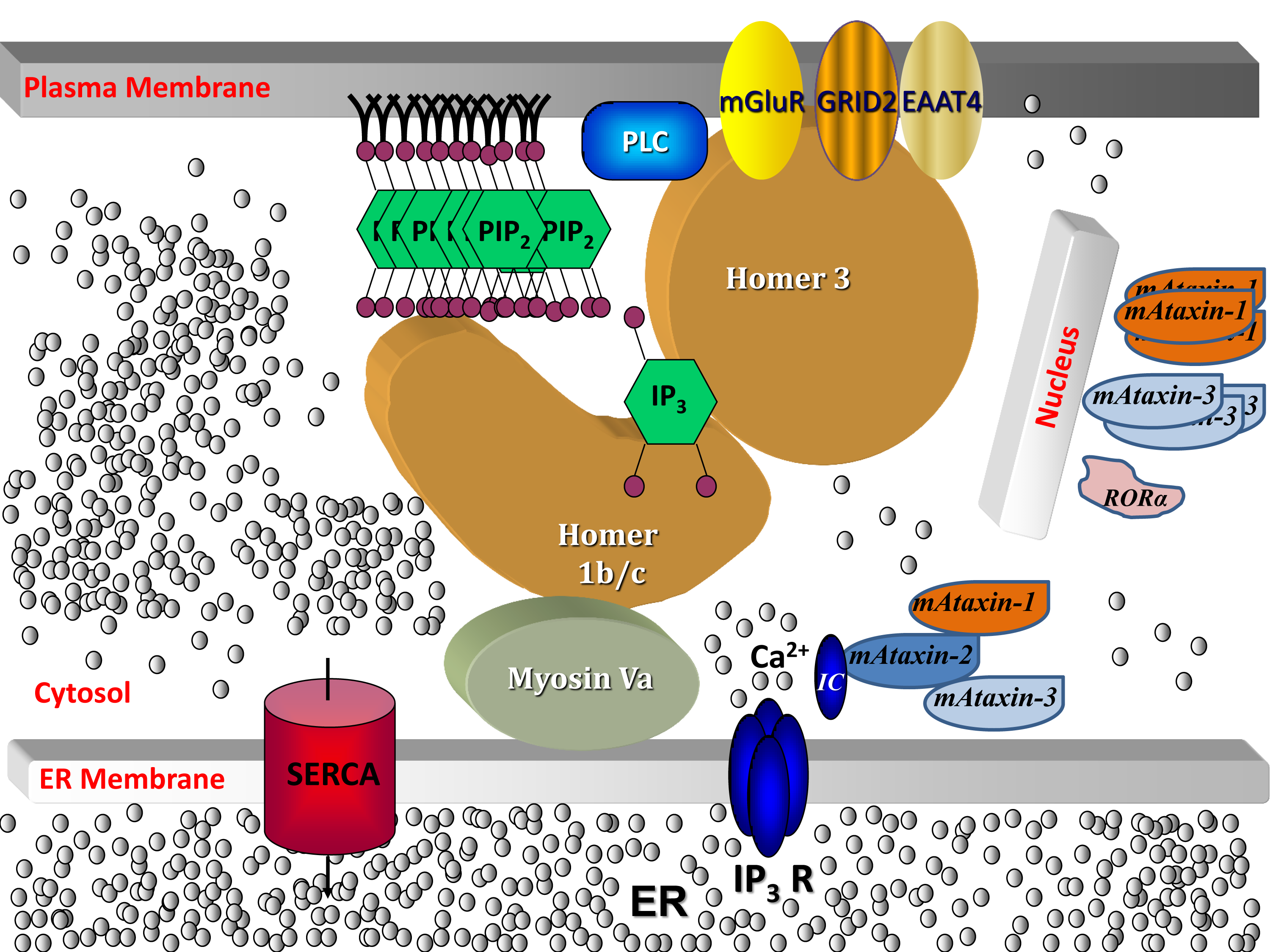

Supplement: Supplementary file 3 [file Image2.TIF]
